# Ablatio-bilica: safety of biliary intraductal radiofrequency ablation in patients with unresectable extrahepatic biliary tract cancer undergoing systemic anti-tumor therapy: a phase II, multi-center, randomized, and controlled study

**DOI:** 10.3389/fonc.2025.1576401

**Published:** 2026-02-24

**Authors:** Tabea Pfister, Samantha Chun Wai Chan, Sven Trelle, Simon Bütikofer, Ralph Winterhalder, Thibaud Koessler, Christoph Schlag, Ralph Fritsch, Jean-Louis Frossard, André Moser, Martin D. Berger, Reiner Wiest

**Affiliations:** 1 University Clinic Visceral Surgery and Medicine, Inselspital Bern, Bern, Switzerland; 2 Department of Clinical Research, University of Bern, Bern, Switzerland; 3 Department of Gastroenterology, Cantonal Hospital Lucerne, Lucerne, Switzerland; 4 Department of Medical Oncology, Cantonal Hospital Lucerne, Lucerne, Switzerland; 5 Department of Oncology, Geneva University Hospital, Geneva, Switzerland; 6 Gastroenterology and Hepatology, University Hospital Zurich, Zuric, Switzerland; 7 Department of Medical Oncology and Hematology, University Hospital Zurich, University of Zurich, Zuric, Switzerland; 8 Department of Gastroenterology, Geneva University Hospital, Geneva, Switzerland; 9 Department of Medical Oncology, Inselspital, Bern University Hospital, University of Bern, Bern, Switzerland

**Keywords:** extrahepatic biliary duct cancers, immune-checkpoint inhibitor, phase II trial, radio frequency ablation, safety

## Abstract

**Background:**

Unresectable and/or metastatic extrahepatic biliary tract cancer (EBTC) presents a clinical challenge with high mortality rates despite therapeutic advancements, for example, chemotherapy + immune checkpoint inhibitors (CICI). One critical aspect is biliary obstruction, which compromises liver function, is associated with complications, and limits the applicability of systemic treatment (chemotherapy with or without ICI). Endoscopic interventions with stent placement alleviate biliary obstruction and, hence, are standard of care. However, stent patency issues and tumor progression remain challenges, prompting the exploration of adjunctive therapies. Biliary radiofrequency ablation (bRFA) induces local tumor destruction, improves stent patency, and potentially boosts the immune response against cancer cells, being synergistic with CICI. Randomized controlled trials (RCT) demonstrated improved overall survival in EBTC but have not been performed in the setting of CICI and have not focused on rate and severity of adverse events (AE). Nonetheless, multiple current meta-analyses propose the use of bRFA in malignant biliary obstruction without high-quality data on its safety in combination with CICI.

**Hypothesis:**

We hypothesize that bRFA in patients with unresectable and/or metastatic EBTC undergoing systemic treatment (chemotherapy with or without immunotherapy) is safe.

**Methods:**

This is a randomized-controlled clinical trial (RCT) comparing chemotherapy with or without ICI plus endoscopic stenting (*n* = 12) versus chemotherapy with or without ICI plus endoscopic stenting and bRFA (*n* = 24) being allocated in a 1:2 ratio. The primary endpoint is the proportion of severe treatment-related adverse events (grade 3 or 4) leading to permanent discontinuation of all active chemotherapeutic drugs up to six months after enrolment.

**Discussion:**

Our findings will provide valuable insights into the role of bRFA as a supplementary treatment in unresectable and/or metastatic EBTC in conjunction with systemic treatment. In case this safety study does indicate no clinically relevant increase in severe adverse events in EBTC treated with systemic treatment, then an RCT addressing the efficacy of bRFA in terms of progression-free and overall survival in this setting will follow.

**Clinical Trial Registration:**

ClinicalTrials.gov, identifier NCT06274879.

## 1 Introduction

Biliary tract cancer (BTC) is the second most common type of hepatobiliary cancer and comprises intrahepatic cholangiocarcinoma (CCA), extrahepatic CCA, and gallbladder cancer. Extrahepatic cholangiocarcinoma/biliary tract cancer (EBTC) can be further divided into perihilar (type III or IV) or distal CCA (type I or II) (1) according to the Bismuth Corlette Classification. EBTC is associated with poor outcomes even when diagnosed at an early stage (2). Overall 5-year mortality is >90%, underlining the need for improvement in clinical management (3, 4).

Standard of care first-line palliative oncological treatment in patients with advanced BTC includes, besides backbone chemotherapy with cisplatin and gemcitabine, immune-checkpoint-inhibition (ICI) with durvalumab (monoclonal PD-L1-antibody) or pembrolizumab (PD-1 antibody) (5–14) 1–10). The phase III randomized, placebo-controlled TOPAZ-1 trial demonstrated improvement in overall survival by durvalumab, improving median overall survival time to 12.8 months as compared to 11.5 months (15). These data led to FDA approval of durvalumab for unresectable and/or metastatic BTC in 2022.

Biliary obstruction significantly impacts survival and quality of life in patients with extrahepatic biliary tract cancer (EBTC) mainly due to associated cholestasis affecting liver function and limiting the effectiveness of chemotherapy due to risks of liver toxicity. Therefore, resolving biliary obstruction as fully and long-term as possible provides broad benefits. The endoscopic standard therapy to relieve and to prevent reoccurrence of biliary obstruction in EBTC is endoscopic retrograde cholangio-pancreatography (ERCP) with biliary stenting. This can be performed either by plastic stents (up to 10 French) or uncovered self-expanding metal stents (uSEMS, usually 10 mm diameter). However, tumor growth into uSEMS can limit stent patency, or tumor extension into more proximal bile ducts can shut off adequate biliary drainage. Given the incurable nature of the disease and the limited duration of stent patency, an additional minimally invasive approach that reduces tumor burden serves several crucial purposes. This approach extends the patency of biliary duct drainage, thereby preventing serious complications such as cholestasis and cholangitis. Furthermore, it facilitates the administration of chemotherapy. Consequently, this strategy holds significant promise for delaying disease progression and enhancing overall survival.

Biliary radiofrequency ablation (bRFA) is a method of minimal-invasive treatment for local destruction of the tumor mass. Based on the results summarized below, bRFA is used more and more in clinical practice (16), with increasing numbers of meta-analyses being published on the subject (17–24). However, none of those meta-analyses provide subgroup analysis on EBTC patients being treated with chemotherapy with or without ICI, and hence, they also do not answer the question on the corresponding safety of this approach in this setting. Nonetheless, bRFA has been demonstrated to improve overall survival in EBTC in multiple case series (16, 25–29) and in three randomized controlled trials (RCT) (30–32). In fact, overall survival as well as stent patency was longer in the RFA group as compared to the control groups (30–32). Finally, RFA patients presented with an improved Karnofsky performance status from 1 through 9 months after the intervention as compared to stent-alone control patients (31, 32). However, all studies were performed in Asia, which is known to differ from Western cases with EBTC in terms of etiology and genetic subtypes. Moreover, those RCTs differed from the proposed investigation either in terms of the cohort recruited, for example, excluding hilar EBTC (31, 32) or using plastic stents (rather than preferable SEMS) (31), or utilizing a different device for RFA (33).

Most importantly, however, no RCT has been performed to evaluate the effect of bRFA in patients undergoing systemic treatment (chemotherapy combined with ICI or chemotherapy alone). Only two retrospective studies addressed the effect of bRFA in combination with chemotherapy (25, 34) but none so far with ICI in palliative EBTC. In both investigations, the bRFA add-on to Gem-Cis significantly improved overall survival [17.3 vs. 8.6 months (25) and 17.1 vs. 11.3 months (34), respectively], as well as progression-free survival [12.9 vs. 5.7 months (25) and 8.6 vs. 5.8 months (34), respectively]. Two RCTs did not observe survival benefit by bRFA in malignant biliary obstruction (35, 36). However, the majority of patients included in those trials had pancreatic cancer with biliary obstruction and/or a minority were treated concomitantly with chemotherapy/checkpoint inhibitors (11, 12).

Safety of treatment is of first-ranked importance during oncological treatments. Adverse events (AE) are critical due to multiple aspects, including treatment acceptance and willingness by the patient as well as their direct impact on patient outcomes via increases in the risk of cancer recurrence/progression by compromising treatment intensity and continuity.

The burden of chemo-immunotherapy–associated adverse events (AE) remains high. In TOPAZ-1, severity grade 3 or 4 AEs according to Common Terminology Criteria for Adverse Events (CTCAE) occurred in 75.7% of the participants in the gemcitabine-cisplatin-durvalumab group and in 77.8% of the participants of the placebo group (gemcitabine-cisplatin-placebo) (37). In fact, 13% of the participants of the gemcitabine-cisplatin-durvalumab group discontinued at least one treatment component due to those AEs. ICI-induced immune-mediated AEs present with a broad portfolio (38), including thyroiditis (37), dermatologic reactions (24, 34, 35, 39, 40), colitis (4), arthritis (37) as well as autoimmune hepatitis (7, 41), and very rare hypophysitis (37). In TOPAZ-1, immune-mediated AEs were observed in 12.7% of the participants in the durvalumab group, and 8% of those AEs were graded as either 3 or 4 according to CTCAE (15, 38).

As for bRFA, most investigations conclude a high safety profile (17–22, 25, 27, 30, 32, 34, 35, 42–44). However, severe complications such as haemobilia (45), choledocho-duodenal fistula (26), cholecystitis (20), and/or hepatic coma (27) have been reported. This underlines the potential safety concern associated with bRFA in EBTC. So far, however, to our knowledge, no prospective and/or RCT has assessed in detail bRFA in the setting of chemotherapy with or without ICI, focusing on its safety and tolerability portfolio. Potential safety concerns relate to tissue damage of surrounding healthy tissue. Moreover, each of the treatment modalities harbors its own potential AE, but combining them may increase the risk of AE. Hence, in the setting of systemic treatment, before planning any RCT, addressing the efficacy of the procedure does need to be assessed prospectively as for its safety.

Therefore, the primary objective of the trial is to provide evidence for the general tolerability and safety of bRFA in patients with unresectable and/or metastatic EBTC undergoing systemic treatment. We hypothesize that bRFA combined with chemotherapy with or without ICI is generally safe and well tolerated by patients. Safety analyses will assess the incidence, nature, and severity of AEs, with clinical and laboratory safety assessments summarized. Overall, the trial methodology aims to rigorously evaluate the safety of the experimental intervention while ensuring statistical robustness and integrity.

The secondary objective of the trial is to gather preliminary data on clinical endpoints, including health-related quality of life and cholestasis symptoms. Additionally, the trial aims to assess procedural aspects such as details about adherence to treatment protocols and total dose of chemotherapy administration as a proxy of safety and tolerability of the experimental treatment. We hypothesize that bRFA benefits by reducing of tumor burden, extending patency of biliary duct drainage, enabling more efficient administration and better toleration of systemic treatment, thereby delaying disease progression and improving quality of life.

## 2 Material and equipment

### 2.1 Chemotherapy immune checkpoint inhibitors therapy (CICI)

Palliative first- and further-line treatments are eligible, as for chemotherapy (with/without checkpoint inhibition), and will be administered in both treatment arms.

### 2.2 Biliary radiofrequency ablation

For standardization of the procedures in this study and to avoid variability of data due to the use of different radiofrequency catheters, the Habib™ probe (EndoHPB Bipolar Radiofrequency Catheter, Boston-Scientific, USA) will be used as the common bRFA catheter. In hilar malignancies, Erbe settings should be limited to 7 Watt since the biliary duct wall is thinner at this site, vascular structures in the adjacent tissue are closer, and hence, complications using higher wattage have been discussed in the literature. If the stricture is more than 24 mm in length, sequential bRFA application will be applied from top to bottom to complete treatment throughout the length of the malignant stricture. Documentation and optimization of bRFA-targeting: Before performing bRFA, the proximal and distal ends of the EBTC should be defined by (i) Radiological imaging, with stored fluoroscopic image depicting the position of the catheter or (ii) Cholangioscopic evaluation accompanied by fluoroscopic imaging, showcasing a stored fluoroscopic image depicting the position of the cholangioscope tip.

**Figure 1:**
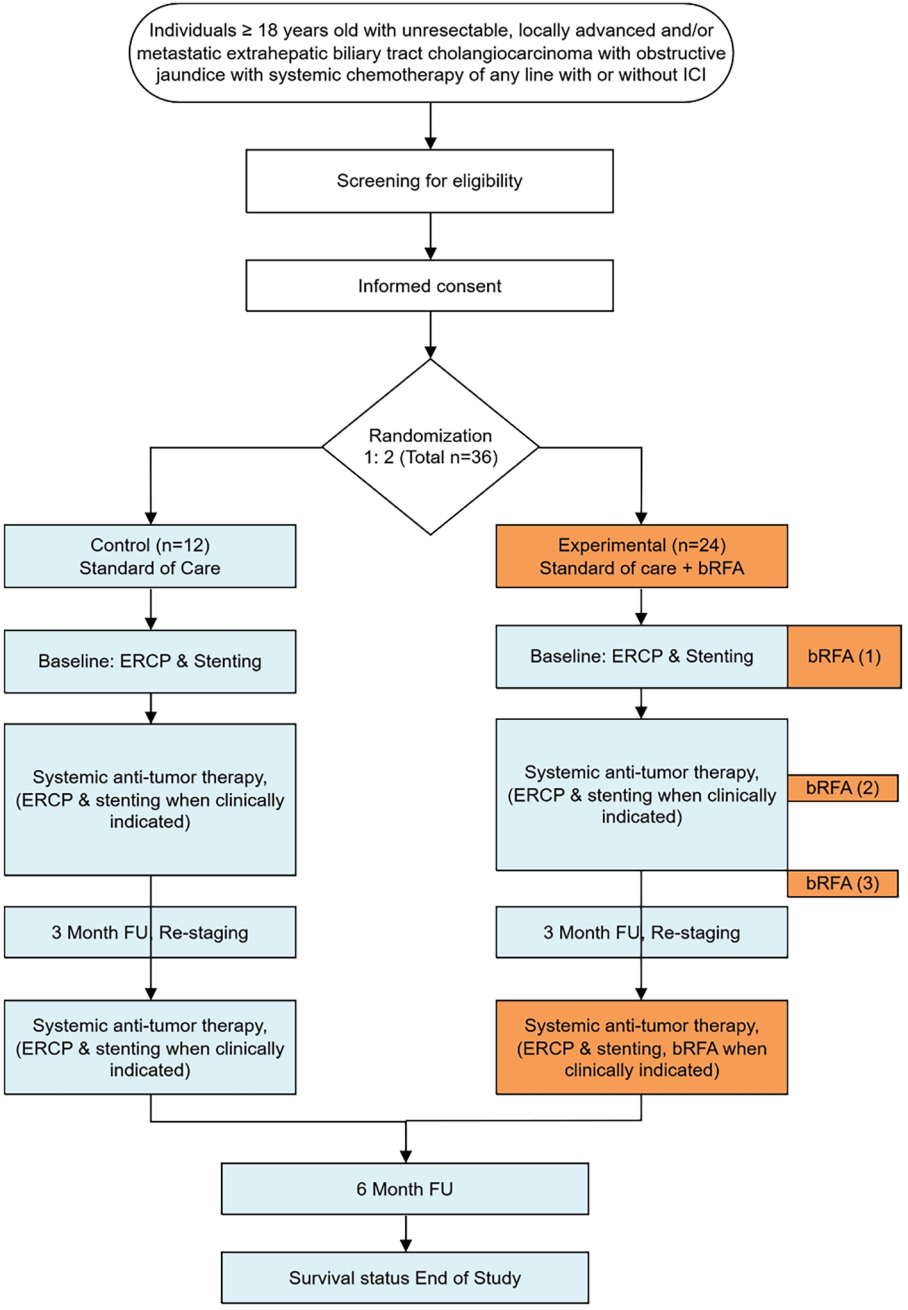
Study design: timeline on the right. Experimental procedure (bRFA) are labelled in orange boxes and blue boxes delineate standard of care treatment.

Biliary stenting: In cases of non-distal EBTC (types III and IV), plastic stents are recommended to achieve biliary drainage. In distal EBTC (types I and II), use of plastic as well as self-expandable metal stents (SEMS) is allowed and the decision is made by the endoscopist. The biliary drainage by prosthesis should:

- Aim to achieve biliary drainage encompassing as much liver volume as possible, aiming for a minimum of over 50% in those patients with impaired liver function
- Avoid drainage of atrophic segments/lobes (e.g., due to portal-vein thrombosis).
- Aim to achieve bilateral stenting in Bismuth class IV cases, as suggested by meta-analysis findings, indicating its superiority over unilateral stenting in lowering hyperbilirubinemia (13) and reducing the risk of re-intervention.
- Be achieved preferentially by plastic stenting facilitating re-interventions and re-treatment.

**Figure 2:**
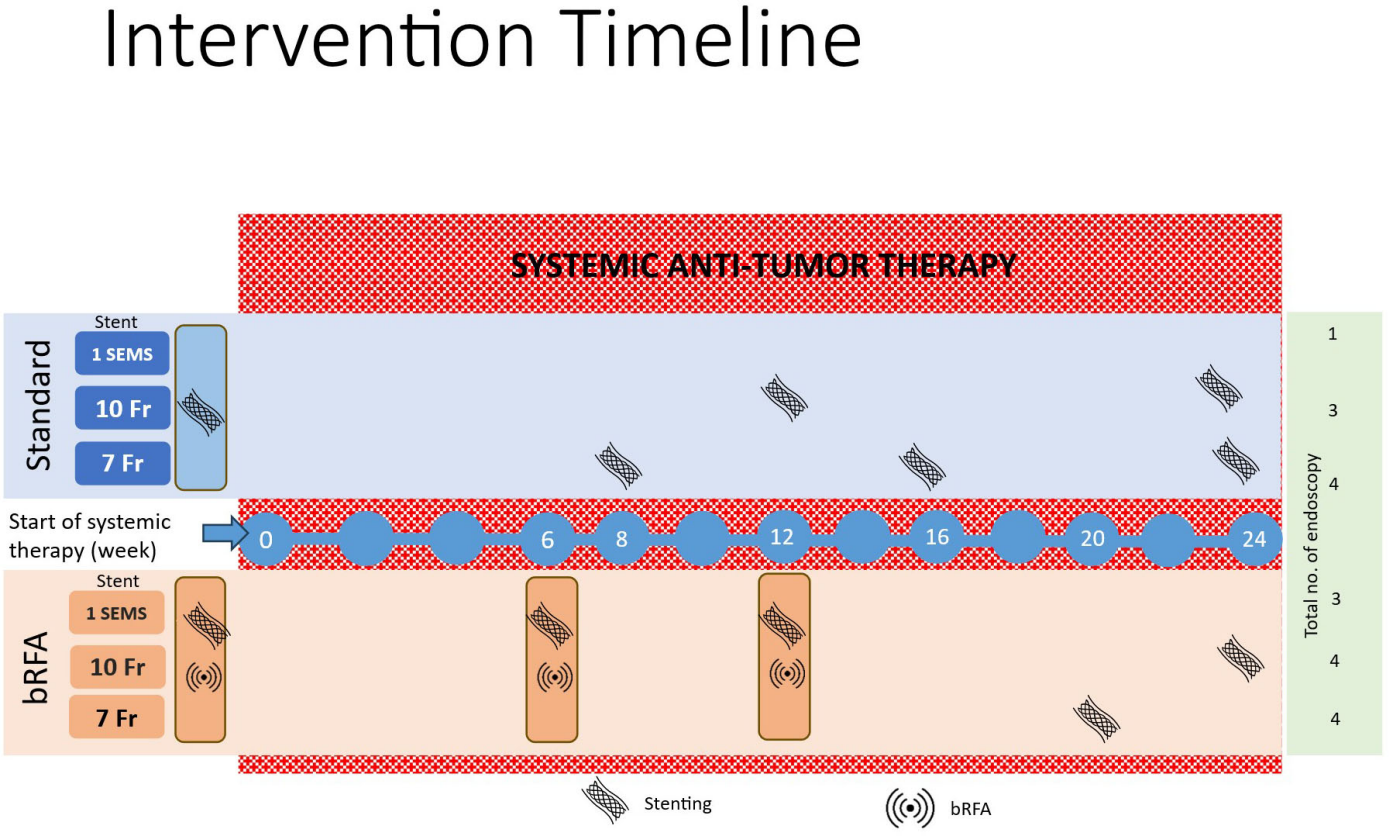
Graphical presentation of the study intervention in the standard group (Standard) and the experimental group (bRFA). The intervention time window of ±4 weeks is allowed for the endoscopy procedure. Examples as for systemic treatment in first line could be CICI or in second line could be FOLFOX initiated at least 24h after stenting and bRFA, if applicable. CICI, *chemo-immune-checkpoint-inhibitor*; Fr, French, indicating the size of the stent; SEMS, *self-expandable metal stent*.

In the case of the use of plastic stents, the interval for reintervention is pre-defined as up to a maximum of 12 weeks for 10 French stents and up to 8 weeks for smaller caliber stents (see **Figure 2**). Documentation of appropriate positioning by saved fluoroscopic image is mandatory.

## 3 Methods

### 3.1 Study design

The study is planned as a randomized-controlled clinical trial comparing systemic anti-tumor treatment (chemotherapy with or without durvalumab plus endoscopic stenting) versus systemic anti-tumor therapy plus intraductal bRFA with a 1:2 allocation ratio. No crossover from standard of care to the investigational procedure is expected during the follow-up period (**Figure 1**).

### 3.2 Patients

Patient selection will be done by standardized optimal diagnostic work-up with (i) cyto-pathological evidenced malignancy, (ii) contrast-enhanced CT or MRI, and (iii) assessment for comorbidities and ECOG-performance status, all based on data obtained from clinical routine procedures. All candidates will be provided with a participant information sheet and informed consent form describing the study.

### 3.3 Inclusion criteria

1. Male or female ≥18 years old.
2. Histologically or cytologically confirmed diagnosis of unresectable*, locally advanced, and/or metastatic extrahepatic biliary tract cholangiocarcinoma with obstructive jaundice (increased serum level of total bilirubin)
3. Cholestasis with bilirubin >3 × ULN (3.6 mg/dl; 63 µmol/L)
4. ECOG performance status ≤2.
5. Adequate bone marrow function: Neutrophil count ≥1.0 × 10^9^/L, platelet count ≥100 x 10^9^/L.
6. Adequate renal function in case of cisplatin administration: Estimated Glomerular Filtration Rate (eGFR) ≥60 ml/min/1.73 m^2^.
7. Willing and able to provide written informed consent.

### 3.4 Exclusion criteria

1. Solely intrahepatic cholangiocarcinoma or mixed-type liver tumors (cholangiocarcinoma with hepatocellular differentiation parts).
2. Multiple hepatic metastases with significant blockage of one or more liver segments and/or less than 50% of liver parenchyma potentially drainable on pre-intervention imaging.
3. In case of ICI administration, any autoimmune diseases, including inflammatory disorders such as Crohn’s disease, ulcerative colitis, Wegener granulomatosis, systemic lupus erythematosus, rheumatoid arthritis, and Graves’ disease.
  (Exceptions: Patients with hypothyroidism following Hashimoto thyroiditis stable on hormone replacement, vitiligo, or any chronic skin disorders that do not require systemic treatment.)
4. Use of immunosuppressive medication within 3 weeks prior to durvalumab administration.
  (Exceptions: Topical or inhaled steroids or systemic corticosteroids at physiologic doses not exceeding 10 mg/day of prednisone or equivalent.)
5. Prior self-expandable metal stent (SEMS) placement in the biliary system
6. Biliary obstruction of non-tumoral etiology.
7. International normalized ratio (INR) > 1.5
8. Secondary tumor (Exceptions: Tumor treated with curative intent without recurrence for more than 5 years or non-melanoma skin cancer, treated carcinoma *in situ* without evidence of disease.
9. Pregnancy or lactation.
10. Known or suspected non-compliance, drug, or alcohol abuse.
11. Inability to follow the procedures of the study, for example, due to language problems, psychological disorders, dementia, and so forth of the candidate.
12. Participation in another interventional study within 30 days prior to enrollment.
13. Other diseases like HIV infection, liver cirrhosis Child-Pugh B or C, history of organ transplantation or condition likely to significantly decrease life expectancy, that is, life expectancy is less than 3 months according to investigator judgement.

### 3.5 Study intervention

Endoscopic treatment: All ERCP (including bRFA procedures in the experimental arm) will be performed by highly experienced endoscopists. These procedures will follow current guidelines (46) and expert recommendations (47). During ERCP, the patient needs to be placed to enable anterior–posterior radiological assessment (either supine or prone but not on the side) to optimize fluoroscopy and associated image analysis. A diagnostic cholangiogram will initially be performed to identify the location and length of the stricture. Both plastic and metal stents can be used, but plastic stents are preferred in this study. Cholangioscopy is performed at each center to retrieve a tissue sample for diagnostic purposes, aiming to visualize the distal and proximal ends of the EBTC and see if the tumor can be passed for comprehensive examination. Prophylactic antibiotics will be administered intravenously, along with rectal administration of indomethacin, for prophylaxis of post-ERC pancreatitis following current guidelines (48).

#### 3.5.1 Definitions used as for: discontinuation of study participation

Participants may be withdrawn from further study participation for the following reasons:

- The participant is unable to begin systemic anti-tumor treatment within 28 days following the baseline ERCP due to not meeting the necessary requirements in 4.4.1.2.
- The participant refuses to continue participation in the study (withdrawal of consent).
- The investigator considers study participation per se harmful for the participant.

#### 3.5.2 Treatment discontinuation

The participant’s systemic treatment, meaning all active antitumoral medication (gemcitabine, cisplatin, and durvalumab), are suspended or discontinued for the following reasons:

- In case of an AE where the investigator considers that patient’s safety may be affected
- In case of a grade 3 or 4 AE (CTCAE V5.0) with sequelae that do not resolve to grade 2 or less, leading to a permanent discontinuation of all active drugs
- Pregnancy

Reintroduction of medication is allowed once improvement or resolution of AE does allow administration following recommendations stated on page 14 delineating CICI dosing.

Technical success is defined as proper stent positioning and confirmation of immediate bile drainage/decompression. Clinical success of jaundice control is defined as a reduction in serum bilirubin by more than 25% after 1 week and by over 50% or reaching a level of ≤50 mmol/L [i.e., ≤3 x Upper Limit of Normal (ULN)] after 1 month, as previously employed in studies concerning this condition (31).

#### 3.5.3 Follow-up endoscopies

The goal of the baseline ERCP is a reduction in serum bilirubin of >25% after 1 week and >50% or ≤50 mmol/L (or ≤3 × ULN) after 1 month. If this is achieved, the endoscopy procedures as individually defined below for each study arm should be followed.

The control arm will undergo routine follow-up with the following criteria for re-endoscopy:

- In the case of plastic stents: After initial stenting, an exchange of the stent(s) is mandatory after a maximum of 12 weeks for 10 French stents and a maximum of 8 weeks for smaller caliber stents. (See **Figure 2**).
- In the case of uSEMS in non-hilar EBTC: Stent exchange should be based on the criteria for re-intervention as outlined in chapter 0.

The experimental arm will undergo pre-scheduled ERCP during the first 3 months to re-evaluate the EBTC-tumor-stenosis and the possibility of re-performing bRFA to ablate the remaining luminal biliary tumor. Hence, a repeat ERCP will be executed every 6 weeks ( ± 4 weeks) after inclusion in the investigation. All unscheduled ERCP and bRFA procedures must be recorded in the unscheduled ERCP eCRF. The endoscopist can decide whether to use plastic stenting or uSEMS for biliary drainage during the ERCP re-intervention at 12 weeks ( ± 4 weeks). Indications for performing bRFA re-intervention are signs of remaining luminal biliary tumor, which can be any of the following observations:

Cholangioscopy with visualization of the remaining tumor at the side of the primary tumor manifestation (where brush and/or biopsies have been taken and/or imaging revealed abnormal biliary wall formation).

Cholangiogram indicates persistent tumor stenosis being defined as at least 25% luminal narrowing compared to the bile duct adjacent to tumor stenosis (if the cholangioscope cannot reach tumor stenosis or is not available).

In the case of previous uSEMS and biliary obstruction due to tumor ingrowth, bRFA will be performed following the same standard operating procedure. Putting in a stent-in-stent add-on is permitted.

##### 3.5.3.1 Criteria for re-intervention outside pre-scheduled regimens for both arms

If clinical success of jaundice control is not achieved by endoscopic intervention, then another attempt to optimize biliary drainage is recommended. Under the following conditions, re-intervention is also allowed:

- Any increase in serum bilirubin by >50% from nadir or >2 ULN (when having been normalized in between) and/or
- Increase in the dilation of intra-/extrahepatic dilatation (above the tumor stenosis) when compared to prior imaging (after endoscopic intervention), and/or
- Any suspicion for cholangitis [defined by Tokyo Guidelines (39, 49–55)—see the Tokyo Guidelines] will trigger suspicion for stent dysfunction (lack of patency). Then endoscopic re-evaluation within 1 week will be performed, and in case of worsened stasis or occluded stent(s), it will be counted as stent dysfunction. The interventional technique to optimize biliary drainage is left to the decision of the endoscopist/operator.

##### 3.5.3.2 Palliative anti-tumor therapy for both arms

Palliative first- or further-line combination chemotherapy will be utilized in both treatment arms and will be administered according to institutional practice and corresponding standard operating procedures (SOP) under the following preconditions:

- >24h after stent placement and, for the experimental group only, bRFA.
- Evidence of jaundice control, defined as a reduction of serum bilirubin >25% after 1 week.
- Bilirubin <6 × ULN and ECOG performance status ≤2.
- Adequate bone marrow function: Neutrophil count ≥1.0 × 10^9^/L, platelet count ≥100 × 10^9^/L.
- Adequate renal function in case of cisplatin administration: Estimated glomerular filtration rate (eGFR) ≥60 ml/min/1.73 m^2^

First-line chemotherapy regimen: Cisplatin/gemcitabine and durvalumab: Each CICI cycle lasts 21 days, where gemcitabine and cisplatin are given on days 1 and 8, and durvalumab is given on day 1. After 21 days, the next CICI cycle starts earliest on day 22 but, if clinically needed, a delay is permitted. The start day of the first cycle of CICI is defined as day 1 of this study, (biweekly administrations). If immune-mediated AEs occur dose delays, and rechallenges are permitted, but no dose reductions are permitted for durvalumab. ICI-related AEs are treated according to the local standard operating procedures (SOP) and are based on the ESMO guidelines (14). In case of contraindications to ICI treatment, patients can be treated with cisplatin and gemcitabine. In case of contraindications for a cisplatin-based regimen, we consider platinum-based agents such as cisplatin, oxaliplatin, and carboplatin as interchangeable and allow their use in combination with gemcitabine and durvalumab or gemcitabine alone at the discretion of the local treating oncologist.

Gemcitabine monotherapy: In elderly, frail, and/or polymorbid patients with an ECOG PS of 2. The treatment schedule can be assessed at the discretion of the treating oncologist (day 1 and day 8 of a 21-day cycle or d1, d8, and d15 of a 28-day cycle or Further-line chemotherapy: for example, second line will follow if feasible the ABC-06 trial utilizing FOLFOX or any oncological drug accredited for treatment of EBTC as decided by the local tumor board according to patient characteristics.

Dose modifications: of the chemotherapeutic drugs (e.g., cisplatin and gemcitabine), including treatment delays or interruptions of the chemotherapy with subsequent rechallenge, are made according to the local standard clinical practice of the treating oncologist.

### 3.6 Outcomes

#### 3.6.1 Primary endpoint

Any grade 3 or 4 AE leading to complete and permanent discontinuation of all active chemotherapeutic drugs (without any re-introduction) up to 6 months after randomization (during the observation period). The decision to withhold systemic treatment will be at the discretion of the treating oncologist according to local SOPs and generally adopted oncological guidelines. AEs will be assessed according to the US National Cancer Institute Common Terminology Criteria for Adverse Events (CTCAE, version 5.0). CICI discontinuation is defined as a permanent and definite stop of all CICI components.

#### 3.6.2 Secondary endpoints, among others, will be

1. Number of CICI courses applied.
2. Total dose of systemic treatment applied.
3. Endoscopic complications according to Adverse events Gastrointestinal Endoscopy (AGREE) criteria (56). Current guidelines are applied for the definitions for cholangitis, post-ERCP pancreatitis, and bleeding.
4. Rate of hospital admissions related to biliary complications, focusing on non-elective endoscopic re-intervention after the first stent placement. This includes details on timing, type of intervention, duration of hospitalization, and resolution of the complication.
5. Death from any cause at the follow-up at 6 months and 24 months (overall survival).
6. Disease-specific quality of life as measured by the EORTC QLQ-BIL21 before every chemotherapy cycle and at 3 and 6 months after enrollment.
7. Health-related quality of life as measured by the EORTC QLQ-C30 at baseline and 3 and 6 months after enrollment

### 3.7 Study duration

The planned overall study duration is 42 months, including the recruitment period of 18 months and a study duration of 24 month follow-up for each participant regarding the survival status or the date and cause of death.

### 3.8 Sample size

The sample size of this study was not calculated based on classical hypothesis testing but on a probabilistic measure, which assesses an excess discontinuation proportion in the experimental arm based on a prespecified sample size of 36 patients (15, 16). The primary endpoint is the proportion of grade 3 or 4 AEs leading to permanent discontinuation of all active chemotherapeutic drugs within 6 months of treatment initiation. We assumed a discontinuation proportion threshold of 10%, which is based on the results of the TOPAZ-1 trial and real-world data (57), respectively. The KEYNOTE 966 reported 3% AE leading to discontinuation (58). We decided to be more conservative and accounted for uncertainty in the discontinuation proportion using a Beta (1.2, 10.8) design prior. The non-safety threshold was set to 20%, which corresponds to a doubling of the assumed discontinuation proportion and is clinically justifiable. Two types of analysis prior distributions were considered for the simulation of trial operating characteristics. First, a skeptical Beta (2.4, 9.6)-prior, which is centered on the non-safety threshold of 20%. The prior parameters were chosen such that the discontinuation probability of 40% is approximately 5%. This led to a prior weight of 12 patients (50% of the planned sample size in the experimental arm). Second, a neutral Beta (0.6, 5.4)-prior, which is centered on the assumed average discontinuation threshold of 10% with a prior weight of six patients (25% of the planned sample size in the experimental arm). After discussion with clinical experts, the skeptical prior was preferred because this prior favors patients’ safety. The neutral prior served as a sensitivity prior for trial operating characteristics. We allowed for early stopping because of non-safety after half of the planned patients. We performed 10,000 Monte Carlo simulations. Simulation results indicated a 21.9% probability of an intolerable trial with the skeptical prior and 11.2% with the neutral prior, while the probability of early stopping due to non-safety with the skeptical prior is 7.7%. Overall, the experimental intervention will be considered intolerable if 6 or more treatment discontinuations due to grade 3 or 4 AEs are reported in the experimental arm, assuming the planned sample size is met under a skeptical prior. If not, the intervention will be deemed tolerable and promising for a phase-III trial.

### 3.9 Randomization

Patient registration and randomization are performed via an internet-based clinical data management system (CDMS), REDCap^®^. Patients are randomized with a block size of 3.

### 3.10 Blinding

Due to the different visit schedules of the two groups, study participants and the treating physicians are not blinded in this study. Moreover, as for the “verum” intervention group with RFA, further study optimization by applying a sham procedure is unrealistic to execute since the same indistinguishable device would be necessary and would not be functioning when being activated (e.g., simulating also the same sound).

### 3.11 Statistical analyses

For the primary analysis of the primary endpoint, we calculate the posterior proportion of CICI discontinuation up to six months using a beta-binomial model with 90% credible intervals using a skeptical beta (2.4, 9.6) prior for both treatment arms. Sensitivity analyses of the primary endpoint will be performed under the neutral beta (0.6, 5.4) prior and a non-informative prior. For secondary endpoints we use conjugate prior models with non-informative priors. Progression-free survival or overall survival will be modeled as a discrete Poisson process using split time intervals as offset. Time will be analyzed using piecewise polynomial splines. We use non-informative centered Gaussian priors with a standard deviation of 1,000 for all fixed effects. We will report modeled survival at specific time points (6 months and 24 months) with 90% credible intervals. Other time-to-event outcomes will be analyzed by Kaplan-Meier curves. Development of EORTC QLQ-BIL21 over time will be modeled using mixed model linear regression.

### 3.12 Interim analysis

One interim analysis will be performed after 12 patients are randomized to the experimental arm. The trial will be stopped early because of non-safety when 4 or more treatment discontinuations because of grade 3 or 4 AEs are reported in the experimental arm. The stopping boundary was calculated based on the predictive distribution under a skeptical prior.

## 4 Anticipated results

We anticipate that the addition of bRFA to standard-of-care systemic anti-tumor therapy and endoscopic stenting will demonstrate an acceptable safety profile, with no significant increase in the incidence of severe treatment-related adverse events (grade 3 or 4) leading to permanent discontinuation of all active chemotherapeutic agents. For that purpose, we implemented a toxicity monitoring design with an unacceptable adverse event threshold of 20%. To favor patients’ safety, we implemented a Bayesian modelling approach with a skeptical prior, which will lead to an early stopping of the trial after 50% of the planned patients. We will stop the trial early if four or more patients in the bRFA arm have permanently stopped all systemic anti-tumor therapy. Based on simulation results, we anticipate a probability of early stopping of 7.7%. At the trial’s end (in total 36 randomized patients), we expect a probability of an intolerable trial of 21.9%. The treatment will be declared as intolerable at the end of the trial if 6 or more patients have stopped all systemic anti-tumor treatment permanently. The used assumptions try to weigh the potential risk profile of bRFA in terms of what can be considered tolerable against the well-known benefits achieved by CICI. The latter, with evidenced survival benefit, should not be offset by an increased rate of discontinuation due to bRFA. This is also the main reason for choosing this primary endpoint since other parameters, although frequently utilized in endoscopic trials on bRFA, such as stent patency, are only indirectly related and most likely to have a low degree of impact on overall survival.

In order to address potential pitfalls, a risk catalogue is made available (“study risk registry”). For this, additional risks that appear atypical in the current study as compared to a “standard” study were identified, rated, and mitigation actions defined (see Appendix 2).

While this study is primarily focusing on safety outcomes, secondary observations may suggest trends toward improved clinical outcomes, such as reduced biliary obstruction-related complications, fewer hospitalizations for stent-related issues, increased dosage of systemic chemotherapy administered and better overall treatment compliance. These results could support the hypothesis that bRFA has the potential to enhance the efficacy of systemic chemotherapy by maintaining biliary function and preventing treatment interruptions.

## 5 Discussion

“Primum non nocere,” or “first do no harm,” is a fundamental principle in medicine emphasizing the importance of ensuring that medical interventions and treatments do not cause unintended injuries and harm to patients. This is true and even more so for palliative conditions, of which unresectable EBTC, despite improvements in treatment, still harbors an unfavorable prognosis. Nonetheless, the TOPAZ-1 trial demonstrated improvement in overall survival by durvalumab (ICI) in addition to standard chemotherapy gemcitabine plus cisplatin (CICI), improving median overall survival time to 12.8 months as compared to 11.5 months (15). bRFA does hold much promise in delivering health benefits to patients suffering unresectable EBTC being treated by CICI, but so far, no RCT has delivered appropriate safety data on its use in combination with CICI or even chemotherapy alone. The study protocol outlined here does serve this purpose and does focus on severe AE leading to permanent discontinuation of all active chemotherapeutic drugs as the primary endpoint. By prioritizing this outcome, the trial provides crucial data on the safety profile of combining bRFA with chemotherapy with or without ICI, an essential first step before exploring efficacy in larger, more comprehensive trials. Additionally, the study employs standardized endoscopic stenting procedures in both groups, offering a controlled environment to evaluate the specific impact of bRFA on stent patency and biliary drainage. This allows for a clearer assessment of bRFA’s role in managing biliary obstruction.

The non-safety threshold of 20%, representing a doubling of the assumed average discontinuation rate of all active chemotherapeutic agents, can be considered as clinically significant and is thought to find acceptance by the oncological community as a minimum safety margin. This reflects the consequence of treatment discontinuation, leaving patients with the best supportive care but no active therapy known to prolong survival. In fact, even survival benefits potentially achieved in those patients left on protocol, in the opinion of the authors, would not justify the utilization of bRFA in combination with CICI in ETBC.

Strengths of the Ablatio-bilica study relate to (a) selection criteria of ETBC patients, (b) standardization of bRFA methodology and (c) study design with systemic treatment and pre-defined repeated bRFA with strict criteria for re-intervention. In more detail, only luminal extrahepatic biliary cancers are selected with exclusion of pancreatic and gallbladder cancer. The latter two also can cause biliary obstruction but most likely represent a different oncological entity with differences in effect size and action of bRFA. Moreover, bRFA is limited to one method, namely, the EndoHabib™ probe (Boston Scientific, USA), which is a plug-and-use instrument with standardized settings and is more commonly used in Swiss hospitals. There are other methods for bRFA on the market, such as ELRA (Taewoong Medical, South Korea) (30, 59), which present differences in technique that could affect safety and hence, were chosen not to be implemented in this protocol. Moreover, on-intention repeated bRFA is pre-defined in the first 3 months and allowed along the course of study, aiming to optimize tumor destruction and hence, efficacy (being assessed in multiple secondary endpoints). This is different from the available RCT data, which usually applies bRFA only once and then places biliary stents without predefined rescheduled ablation sessions. Available data on regular additional bRFA in unresectable ETBC do indicate a potential add-on benefit by this approach (25), with case reports on long-term survival extending to 72 months. Indeed, bRFA combined with chemotherapy in a real-life cohort of advanced ETBC resulted in significantly longer median overall survival (17.3 vs. 8.6 months). In terms of safety, no difference in severe hematological toxicities (CTCAE grades 3–5) was detected between groups, whereas therapy-related cholangitis occurred more often in the bRFA group (25). However, this trial was retrospective in design, and thus, no high quality bias-free statement as for safety can be drawn. Finally, no immune-checkpoint inhibitor was applied, and thus, considering CICI the optimal standard-of-care treatment, any additional interventional approach does need to prove its safety in conjunction with systemic treatment in a prospective trial.

This study has some potential limitations. First, the small sample size of 36 patients (12 in the control group and 24 in the experimental group) may limit the statistical power to detect rare adverse events or more subtle differences between the study groups. Second, differences in the composition of the chemotherapy may have an influence on the safety and tolerability of bRFA in the experimental arm. Another limitation is the potential for patient selection bias. Lack of stratification due to the limited sample size per study group may inadvertently favor patients with a less advanced disease, limiting the generalizability of the findings to the broader population of patients with unresectable biliary tract cancer. Moreover, the heterogeneity of biliary tract cancers—encompassing a variety of tumor types with distinct biological behaviors—could introduce variability in treatment responses. The study also faces challenges related to operator dependency. The success of bRFA and stent placement can vary depending on the skill and experience of the endoscopist.

Nonetheless, we are confident that Ablatio-bilica represents an important step towards refining and improving treatment strategies for unresectable EBTC. The study’s findings, if positive, will lay a solid foundation for further research into the long-term benefits of bRFA in combination with systemic chemotherapy in advanced ETBC. In fact, the generated evidence by Ablatio-bilica will then help to design a confirmatory RCT with a larger sample size and a relevant efficacy endpoint (overall survival) in patients suffering unresectable EBTC being treated by systemic treatment in the first- or second line setting.

## Data Availability

The original contributions presented in the study are included in the article/supplementary material. Further inquiries can be directed to the corresponding author.

## Ablatio-BiliCa-Consortium

C. Schlag, Department of Gastroenterology and Hepatology, University Hospital Zurich, Zurich, Switzerland; R. Fritsch, Department of Medical Oncology and Hematology, University Hospital Zurich, University of Zurich, Zurich, Switzerland; JL Frossard Department of Gastroenterology, Geneva University Hospital, Geneva, Switzerland T. Koessler, Department of Oncology, Geneva University Hospital, Geneva, Switzerland.
